# Navigating the complexity of p53-DNA binding: implications for cancer therapy

**DOI:** 10.1007/s12551-024-01207-4

**Published:** 2024-07-11

**Authors:** Kelly M. Thayer, Sean Stetson, Fernando Caballero, Christopher Chiu, In Sub Mark Han

**Affiliations:** 1 College of Integrative Sciences, Wesleyan University, Middletown, CT 06457 USA; 2 Department of Chemistry, Wesleyan University, Middletown, CT 06457 USA; 3 Department of Mathematics and Computer Science, Wesleyan University, Middletown, CT 06457 USA; 4 Molecular Biophysics Program, Wesleyan University, Middletown, CT 06457 USA

**Keywords:** p53, Allostery, Drug design, Machine learning, MD-MSM, MD sector, Graph theory

## Abstract

**Abstract:**

The tumor suppressor protein p53, a transcription factor playing a key role in cancer prevention, interacts with DNA as its primary means of determining cell fate in the event of DNA damage. When it becomes mutated, it opens damaged cells to the possibility of reproducing unchecked, which can lead to formation of cancerous tumors. Despite its critical role, therapies at the molecular level to restore p53 native function remain elusive, due to its complex nature. Nevertheless, considerable information has been amassed, and new means of investigating the problem have become available.

**Objectives:**

We consider structural, biophysical, and bioinformatic insights and their implications for the role of direct and indirect readout and how they contribute to binding site recognition, particularly those of low consensus. We then pivot to consider advances in computational approaches to drug discovery.

**Materials and methods:**

We have conducted a review of recent literature pertinent to the p53 protein.

**Results:**

Considerable literature corroborates the idea that p53 is a complex allosteric protein that discriminates its binding sites not only via consensus sequence through direct H-bond contacts, but also a complex combination of factors involving the flexibility of the binding site. New computational methods have emerged capable of capturing such information, which can then be utilized as input to machine learning algorithms towards the goal of more intelligent and efficient de novo allosteric drug design.

**Conclusions:**

Recent improvements in machine learning coupled with graph theory and sector analysis hold promise for advances to more intelligently design allosteric effectors that may be able to restore native p53-DNA binding activity to mutant proteins.

**Clinical relevance:**

The ideas brought to light by this review constitute a significant advance that can be applied to ongoing biophysical studies of drugs for p53, paving the way for the continued development of new methodologies for allosteric drugs. Our discoveries hold promise to provide molecular therapeutics which restore p53 native activity, thereby offering new insights for cancer therapies.

**Graphical Abstract:**

**Figure:**
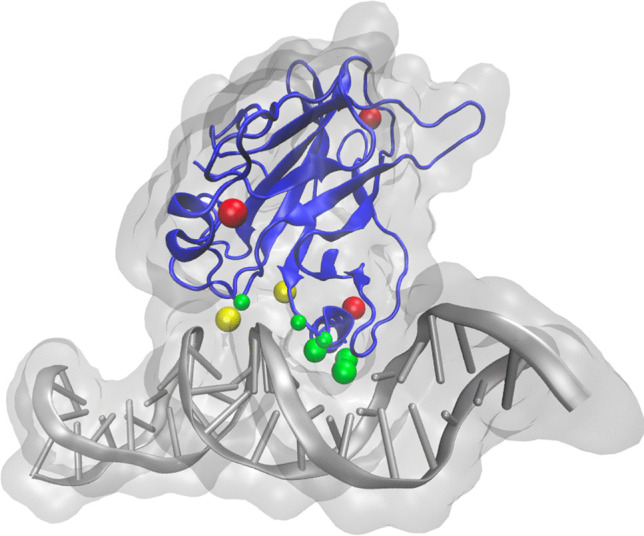
Structural representation of the p53 DBD (PDBID 1TUP). DNA consensus sequence is shown in gray, and the protein is shown in blue. Red beads indicate hotspot residue mutations, green beads represent DNA interacting residues, and yellow beads represent both

## Introduction

The tumor suppressor protein p53 is at the forefront of preventing cancers mainly through its interaction with DNA. When the cell has suffered an insult such as DNA damage, its primary role is to determine cell fate, either cellular repair or apoptosis, both with the goal of removing faulty cells from the gene pool. Mutation of p53 leaves compromised cells vulnerable to proliferate unchecked, leading to cancerous tumors. An estimated 50% or more of all human cancers are attributable to a mutation in this protein alone (Vogelstein et al. 2000), a fact which has earned it the title “Guardian of the Genome” (Lane 1992). Cancer is regarded as the second leading cause of death globally, accounting for about 9.6 million deaths, or just under 20% of all mortalities in recent years (Islami et al. 2021; Sung et al. 2021; Wild and Stewart 2014). The importance of curing cancer or at least advancing treatments is thus plainly evident.

While many labs and even entire institutions engaging the foremost scientists of our time work to find increasingly effective ways of dealing with the illness, surprisingly few advances for molecular-based therapies for curing cancer have emerged with any degree of success. While p53 is a complicated protein acting as a single, albeit important, hub in a wide regulatory network (Vogelstein et al. 2000), its direct control of genes can be cast in a simplistic view accessible to molecular biophysicists: it is a transcription factor binding to DNA, and when mutations occur, changes in the ensemble of accessible structures change, resulting in incorrect binding (Lane 1992). The challenges for molecular biophysicists to contribute to this field lie in the areas of understanding allostery; studying how a change far from the DNA binding interface can cause a change in the binding, and how to reinstate the native activity through drug design. New advances in graph theory (Abramson 2021; Novak and Gibbons 1999; Prathik et al. 2016) and networks (Aggarwal et al. 2021; Han et al. 2022; Novak and Gibbons 1999) can be applied to biomolecules to consider how mutated molecules may react. Additionally, machine learning techniques can discern subtle allosteric signals amidst large datasets. These methods can for the first time be applied to biomolecular problems using molecular simulations. In this review, we consider the current state of understanding of the structure and function of p53. We then proceed to understand how mutations may disrupt crucial interactions, and what efforts have been made at the molecular level to restore activity. Finally, we consider cutting-edge ideas in the field coupled with recent advances in graph theory and machine learning, which we envision as an exciting and novel path forward to open new possibilities to design molecular-based therapeutics to restore p53 activity. It is an urgent and timely engineering problem requiring the cooperation of those in many disciplines. We consider this in light of p53, while realizing the possibility for translational information to impact the broader engineering and drug design fields to not only positively impact numerous currently undruggable diseases, but also as a means of allosterically designing the function of molecules.

## p53 structure

### Insights from the first crystal structure

Much of the insight into the mechanistic working of p53 has come from structural studies, most notably starting with X-ray crystallography. p53 is a 393 amino acid protein encoded by the TP53 gene which was discovered in 1979. It was identified as a target of SV40 tumor antigen (Linzer and Levine 1979); soon after, it was cloned and sequenced (Zakut-Houri et al. 1985). The full-length protein can be considered in terms of functional domains: the transcriptional activation domain (TAD, residues 1–62), which comprises a little over half of the N-terminal intrinsically disordered region of the protein; a proline-rich region (residues 63–69); the DNA binding domain (DBD, residues 102–292); a tetramerization/oligomerization domain (TD, residues 320–360); and the intrinsically disordered C-terminal (residues 361–393).

The proline-rich region contains an especially high number of proline amino acids, noted as the only amino acid whose side chain loops back to the backbone, giving it unique backbone angle accessibility readily identified in Ramachandran plots. This region has been associated with the apoptotic activity of p53, but again how this intrinsically disordered region confers activity is unexplained (Baptiste et al. 2002).

The DNA binding domain intersperses between the two intrinsically disordered regions. It forms a well-structured albeit thermally tenuous beta-barrel (Butler and Loh 2006). The tetramerization domain is believed to facilitate the quaternary association of four monomers to form a tetrameric structure, and a chemically basic region in the remainder of the C-terminus (Hamard et al. 2012; Jeffrey et al. 1995). The C-terminal region regulates p53 between a latent and active state where p53 is inactive and active for DNA binding, respectively, though the exact mechanism remains unclear (Ayed et al. 2001). Post-translational modifications to the C-terminal region modulate DNA binding affinity; p53 isoforms without the C-terminal region have higher specific sequence binding affinity (Appella and Anderson 2001; Ayed et al. 2001; Gu and Zhu 2012). While the C terminus is well established in receiving PTMs, how these regulate the DBD remains largely unclear in light of NMR evidence suggesting no conformational difference in the presence or absence of the C-terminal region (Ayed et al. 2001; Hamard et al. 2013).

Intrinsically disordered regions (IDR) of proteins (Oldfield and Dunker 2014) are highly mobile parts evading conventional structural determination. However, they bear considerable biological importance. In p53 they comprise about half of the protein and are the major sites of post-translational modification (PTM) regulation (Gu and Zhu 2012; Hamard et al. 2013; Oldfield and Dunker 2014). Despite lacking static structure, IDRs have a multi-funnel energy landscape (Chebaro et al. 2015), indicating that they can adopt specific structures, similar to the ordered nature of a static structure, but they more broadly sample conformational space. Although the dynamic nature of IDRs makes them largely inaccessible to experimentation, they may have been selected by nature as versatile control switches with multiple rather than binary options (Oldfield and Dunker 2014), as seems to be the case in p53. How the interdomain communication works from an intrinsically disordered region to the DBD remains largely unexplored.

**Fig. 1:**
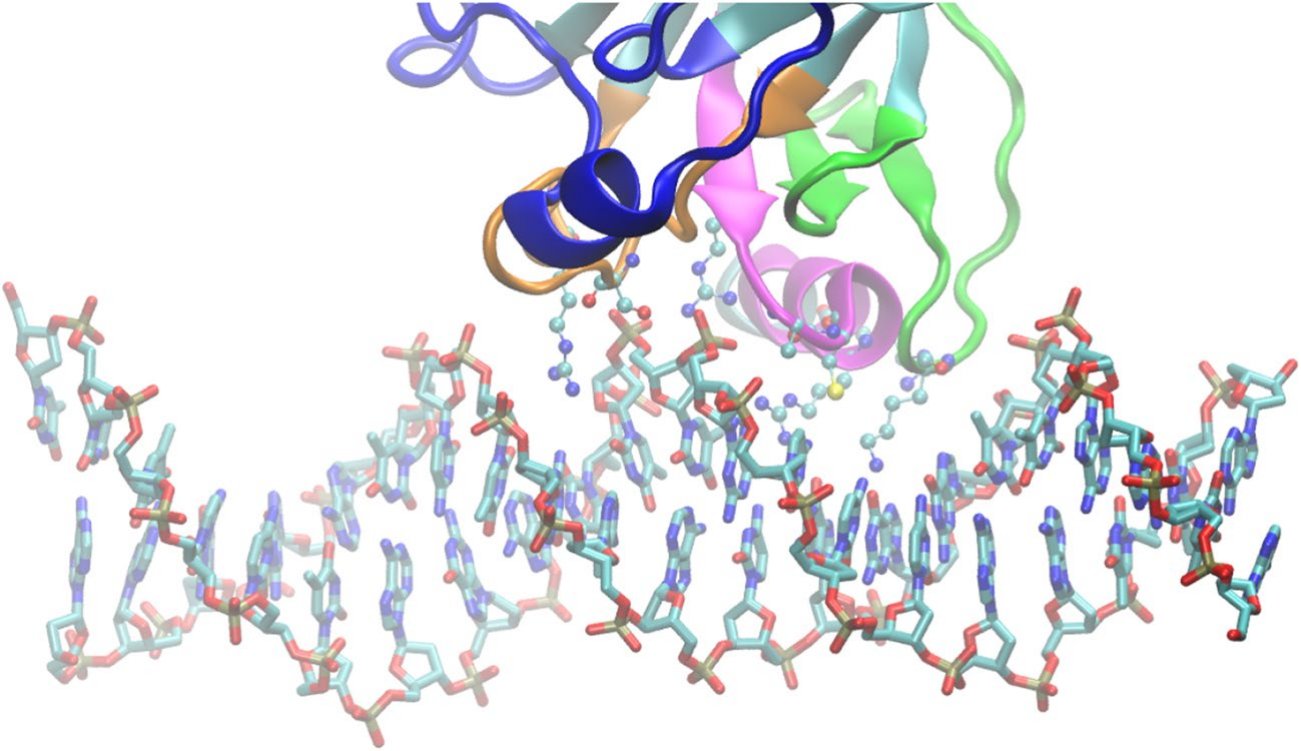
DNA interaction with p53 protein. Important structural elements involved with the DNA binding are highlighted: the L1 loop (residues 113–140, green), the L2 loop (residues 165–195, royal blue), the L3 loop (residues 236–251, orange), and the strand loop helix (residues 271–286, magenta). The residues K120, R241, K248, K273, A276, A277, R280, and R283 H-bonding with the DNA are displayed in CPK ball and stick representation

The core of the protein was first crystallized in 1994 (PDBID 1TUP) by Cho et al. (1994) providing considerable atomic level detail of the structure of the protein and its interaction with an engineered consensus DNA sequence. Within the DBD, p53 exhibits classic beta barrel structure, along with some key structural features. In addition to the beta sheets making up the beta barrel, it contains a variety of important structural elements involved with DNA binding: the L1 loop, the L2 loop, the L3 loop, and the strand loop helix (Fig. 1B). Furthermore, it chelates a zinc via four coordination residues C176, H179, C238, and C242 which are located in the loops L2 and L3 (Blanden et al. 2015). The zinc is obligate; without these tether points, the structural integrity collapses in a short period of time, specificity is lost, and p53 tends to aggregate (Butler and Loh 2003).

## H-bond interaction surface

Furthermore, the 1TUP structure (Fig. 1) (Cho et al. 1994) (2.2 angstroms resolution, R factor of 20.5%) identifies eight residues directly H-bonding with the DNA: K120, R241, K248, K273, A276, A277, R280, and R283. To facilitate crystallization, a sequence of high consensus was used instead of a biological sequence, which, as shall be addressed below, tend to deviate from the more optimally interacting consensus by having fewer H-bonds between the protein and DNA. In this binding interface, six of the eight interactions mediate protein side chain H-bond interactions to the DNA backbone: K120, S241, R248, R273, A276, R283. Only three residues, K120, C277, and R80, have capabilities of making discriminate interactions occurring in the DNA major groove. K120 falls in both categories due to the formation of two distinct H-bonds. In addition to studying p53 bound to DNA, the corresponding unbound version is also available (PDBID 2OJC) (Wang et al. 2007). The crystal structure preempts a major question in the p53 field: only three positions of the binding site—the decamer helix directly interacting with protein side chains—appear to have sequence discrimination, so how is p53 able to identify binding sites? This question will be revisited in the forthcoming sections.

## K120 alternate conformations

Further crystallographic studies captured two distinct conformations of the L1 loop leading to differences in K120 binding to DNA (Petty et al. 2011). Interestingly, these were captured within the same crystal; because of the space group of the crystal, two different p53 monomers were present within the same unit cell. The extended conformation and the recessed conformation in chains B and D respectively (PDB ID 3Q05) both appear under the same environmental conditions, suggesting that the two conformations may be energetically interchangeable. Further investigation suggested that the loop appears to primarily form the extended conformation when bound as a monomer and when free in solution. The recessed form again appears in a tetrameric form bound to DNA (PDB ID 4MZR) in a study of an engineered sequence (Emamzadah et al. 2014).

## Hotspots

p53 is known to have several positions that, when mutated, have an exceptionally high prevalence in cancerous samples (Fig. 1). Such data has been collected by sequencing of p53 originating from tissue samples from human cancer patients and is curated by the IARC database (Hernandez-Boussard et al. 1999; Olivier et al. 2002) as an ongoing project. The following ten mutations have emerged as the top frequency hotspots: R175H, R248Q, R273H, R248W, R175L, Y220C, R273C, R282W, R248L, R175P. Of these, the top six account for 30% of all mutations. Interestingly, these mutations localize to the DBD. A potential explanation for this is offered in the following section. Although the Y220C mutation is not in the most frequent group, it is significant for its role in drug discovery; namely, the drug PK11000 has been shown to restore wild-type function in the Y220C mutation in vivo, eliminating cancerous tumors (Bauer et al. 2016; Zhou et al. 2023).

### p53 oligomerization, quaternary structure, and the tetramerization domain

**Fig. 2:**
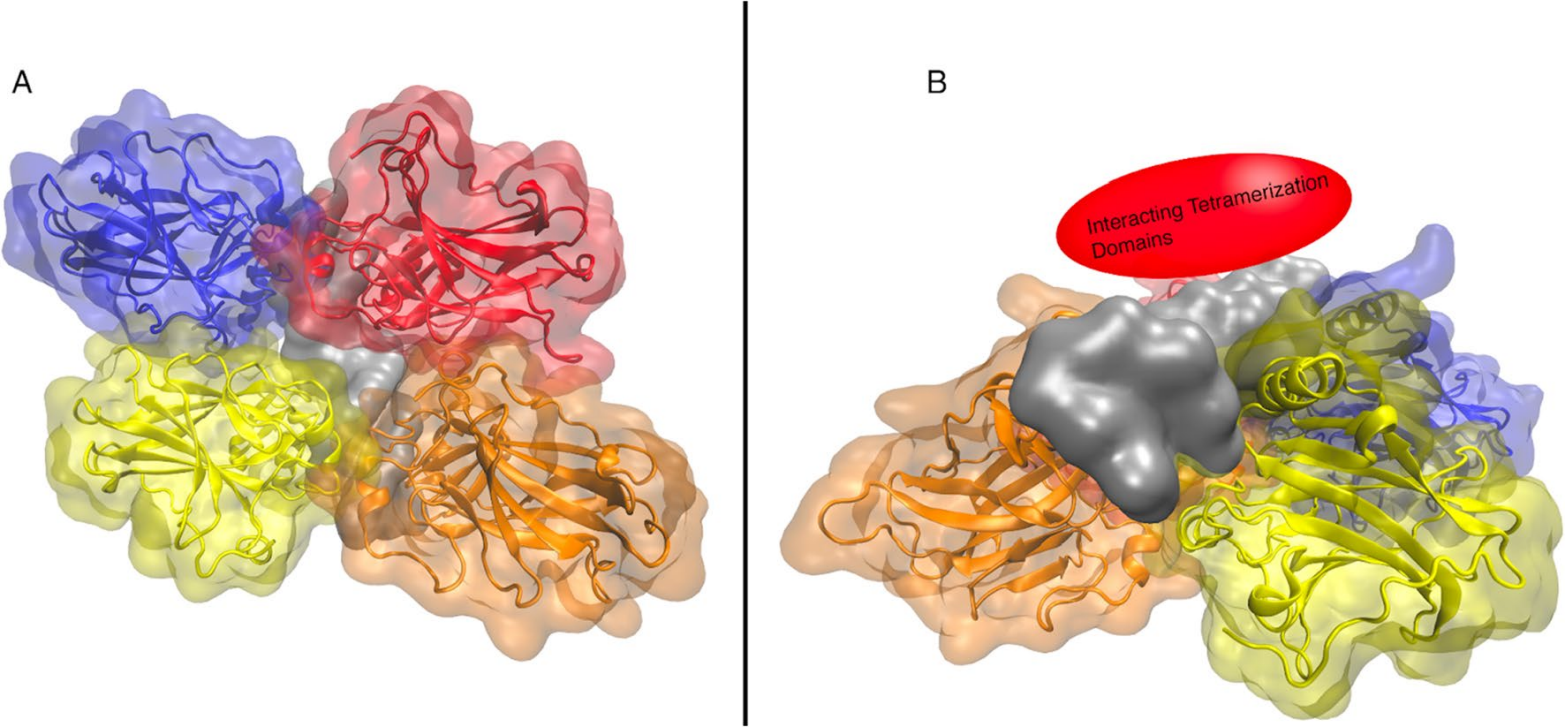
PDBID 3KMD. **A** Structural diagram of a p53 tetramer. Each p53 monomer is shown in a different color, and DNA is in gray. **B** The same diagram but from a different angle, showing where the four interacting TDs would be in relation to the DBDs and the DNA

Similar to many transcription factors, p53 can oligomerize to form quaternary structures (Fig. 2). This is facilitated through the tetramerization domain (TD), which, as noted above, is found in the disordered C terminal region of p53. It has been crystallized in the form of four fragments (Jeffrey et al. 1995). Isoforms of p53 lacking the TD are still able to bind to DNA and initiate transcription. However, the binding affinity of isoforms without the TD is 10 to 100 times lower than those with a TD (McLure and Lee 1998). Tetramerization of p53 is required for at least some functions of p53. For example, ubiquitination—a universal signal for nuclear export and localization to the proteasome for destruction—of p53 requires it to be oligomerized; the nuclear export signal exists between residues 340 and 351, which is only exposed when p53 is monomeric and buried when it is tetramerized.

The TD is highly sensitive to mutations due to compounding destabilizing effects between four copies of the protein. Thus, even a single mutation in each of the four TDs is highly likely to at least weaken if not totally inhibit oligomerization. For this reason, a mutation in the TD may be as devastating to p53 wild-type (WT) function as a mutation in the DBD is, but as mentioned above, TD mutations do not number among the cancerous hotspot mutations. Many p53 gain-of-function mutations that result in cancer require oligomerization and the function of the TD and/or the C-terminal region; consequently, few cancers are found to be caused by point mutations in the TD which would inhibit oligomerization (Chène 2001).

While the tetrameric form is generally thought to be the biological form, there is controversy on the matter (Okorokov and Orlova 2009). Two structural studies contrast in the relative orientation of the monomers which leads to differences in the relative position of the binding interfaces. The first model, from X-ray crystallography, purports the monomers lay on the DNA in a head-to-head fashion, such that they contact the DNA in succession (Kitayner et al. 2010, 2006). The consensus sequence is made up of two half-sites, which are themselves made up of two head-to-head quarter sites (→ ←  → ←) (McLure and Lee 1998). It follows that when p53 does bind to the DNA consensus sequence as a tetramer, it does so as a dimer of dimer of p53 monomers. The monomer-dimers bind at consecutive quarter sites, rather than alternating quarter sites.

To further confuse matters, one DNA half-site appears to be sufficient in binding to the consensus sequence (Ly et al. 2020), but the presence and simultaneous bonding of a second dimer in the tetramer has been reported to increase the binding affinity by 50-fold (McLure and Lee 1998). This, however, is somewhat surprising when juxtaposed with a genomic ChIP assay in which monomeric sites were often sufficient within the genomic DNA for competitive binding and subsequent immunoprecipitation.

In contrast to the crystallographic head-to-head model, the CryoEM structure of the full-length p53 at 13.7 angstrom resolution best characterizes p53 as two oppositely arranged dimers (Okorokov et al. 2006) (PDB: 1igt). This opens the possibility that p53 could bind DNA at two distinct interfaces on opposite sides, either as two distinct strands being bound simultaneously or a single strand that is bent back on itself to thread through twice. It is also possible that p53 could be capable of both of these structures, or even more, or bind in complexes, under different biological conditions.

Nevertheless, a binding interface involving a tetramer may confer overall improved binding for the complex in a kind of cooperativity. There are at least three plausible explanations for this increased binding affinity. First, interactions between the four TDs create a conformational change that enhances binding, and second, the presence of a second dimer simply increases the overall enthalpic interaction the tetramer has on the DNA. Third, dimer–dimer interactions increase the likelihood of the tetramer finding a better consensus sequence than just a half site. The p53 tetramer would randomly sample DNA conformations, isolated quarter-sites having low binding affinity and half-sites having a higher binding affintiy. This would ensure that the tetramer would be able to sufficiently sample enough conformations at a consensus sequence and increase the likelihood of a successful binding. This complex would be reinforced with dimer–dimer interactions (McLure and Lee 1998). It is essentially a thermodynamic argument in which the binding of one monomer decreases the configurational search space for subsequent attached monomers to locate DNA to bind.

### Response elements

A response element can be defined as nucleotide sequences recognized by regulatory transcription factors, which then lead to gene response to various regulatory elements. p53, serving as a transcription factor binding to defined DNA sites, activates gene expressions in stress response, which can vary from cell cycle control, DNA repair, and apoptosis. p53 creates complexes with either consensus or natural response elements, with the complexes having similar binding affinity and specificity, as confirmed by binding competition against bulk genomic DNA (Vyas et al. 2017). Moreover, a question that remains unsolved is how p53 efficiently manages its functions in response to stress, given that the p53 target genes, involved in early stress response pathways, possess more flexible response elements when compared to genes which require strict regulation or those genes with outcomes happening later in stress responses. The flexibility of response elements contribute to the expression of p53 target genes, influencing the decisions of the system (Safieh et al. 2021).

### p53–non-B-DNA interactions

Keeping in mind that p53 operates in the nucleus under conditions of DNA damage, it is not surprising that it can bind to alternative DNA conformations that abound in the nuclear environment. A typical cellular nucleus is about 5 to 20 µm in diameter, housing approximately 2 m (about 6 feet) of DNA. This feat is achieved by the wrapping of DNA around nucleosomes and interactions with other proteins that result in its supercoiling. As a result, much of this DNA is not in the canonical B-form (Corless and Gilbert 2016; Ravichandran et al. 2019); it may be in different double-stranded forms, such as A-DNA, C-DNA, or Z-DNA; there may be additionally a variety of other non-canonical forms encountered as the cell undergoes processes such as transcription and replication. While the DBD is responsible for making most B-DNA interactions, as well as Z-DNA interactions, triplex and G-quadruplex DNA interacts with C-terminal residues of p53. Hotspot mutations in the DBD cause conformational changes or directly interfere with the DNA binding interface, abolishing the ability of p53 to recognize and bind to DNA sequences. p53 exhibits structure-specific DNA binding properties; it has the ability to bind a wide array of DNA structures including positively and negatively supercoiled DNA, three-way and four-way junctions, telomere T-loops, hemicatenate DNA, and cruciforms (Brázda and Fojta 2019; Jagelska et al. 2010; Štros et al. 2004). Hemicatenate DNA (Štros et al. 2004) is an intermediate structure formed during DNA replication, repair, and recombination. Their primary feature is that one of the strands is longer than its partner(s), and hemicatenate DNA can be found in single-stranded, double-helical, or four-stranded DNA. p53 has been shown in vitro to bind to hemicatenate DNA, and can adopt one of three different complexes, suggesting that p53 has the ability to differentiate between types of hemicatenate DNA.

Sequences of mirror-symmetric homopurine homopyrimidine DNA strands have been shown to form triple-stranded structures. G/C-rich sequences have been shown to form two types of four-stranded structures: G-quadruplexes and i-motifs. Both triplex and quadruplex structures occur non-randomly throughout the human genome; triplex sequences inhibit transcriptional regulation, and G-quadruplex structures are significant in DNA replication, telomere maintenance, and transcriptional regulation. p53 has been shown to have high affinity for binding to triplex and G-quadruplex structures (Adámik et al. 2016).

### Crystallographic plausibility argument for molecular-based drug design

Crystallographic studies have also shed insight into the possibility of rational drug design to restore the activity of p53 mutants. The mutations R249S and T123A together are known to disrupt protein activity (Joerger et al. 2005; Nikolova et al. 2000; Suad et al. 2009). It has been reported that the distal H168R is capable of restoring normal p53 binding for these joint mutations. The study illustrates the principle that a mutation can be “undone” by the introduction of another perturbation to the system.

Other work has focused on the Y220C mutation site; while it is not a top-ranking hotspot, it has been a focus of study due to its prominent role in p53 restoration studies. One key feature of this mutation is a loss of an aromatic ring, and thus, one might reason a strategy to reverse the problem and restore the WT function would focus on the reintroduction of the aromatic back into the system as a sort of molecular prosthetic. To this end, compounds such as PK083, PK7088, and PK11000 emerged (Bykov et al. 2018, p. 53; Lopes et al. 2019; Stahlecker et al. 2022). PK083 and PK7088 did not yield the desired results, but PK11000 did in an unexpected way: rather than locating at the site of the mutation, PK11000 was discovered to bind covalently at 182, distal from both the mutation site and from the DNA (Bauer et al. 2016). In rodent models, it convincingly eradicated tumors involving Y220C (Bauer et al. 2016). Unfortunately, it has not been able to pass clinical trials, likely due to side effects from indiscriminately covalently binding to off-target thiols such as solvent-accessible cysteines of other proteins. These advances have hinted at the possibility that an active site of a transcription factor such as p53 could be modulated by using principles of allostery.

## Biochemical and bioinformatics studies investigate p53 indirect readout mechanism for site discrimination

Biochemical and bioinformatics studies have also substantially contributed to the structure and function knowledge of this protein. Up to this point, several lines of evidence have begun to hint that p53 binding to target DNA may not follow a simple predictable lock and key mechanism.

As with many transcription factors, p53 binds a variety of DNA binding sites for which a general pattern emerges. The general consensus for p53 binding sites is 5′-RRRCWWGYYY-3′ where R = purine, Y = pyrimidine, W = A or T, and C and G represent their respective DNA bases (El-Deiry et al. 1992). This is consistent with the sequence of DNA in the 1TUP crystal structure (Cho et al. 1994). Such a sequence optimally complements the available H-bond donor and acceptor groups. In fact, simple models for computing the binding affinity of p53 to linear B-DNA sequences have been created (Brázda et al. 2016; Veprintsev and Fersht 2008).

Nevertheless, chromatin immunoprecipitation data suggests that p53 actually binds to a surprisingly wide array of sequences within the genomic context. In brief, the assay presents p53 with many genome fragments, which compete with each other to be bound by p53. Bound p53 is immunoprecipitated out and the DNA is extracted, then amplified and sequenced. Thus, a selection for sequences preferentially bound by p53 is obtained. Rounds of competitive binding, called SELEX, can also be applied to obtain an ever-increasingly selective binding pool (El-Deiry et al. 1992; Qian et al. 2002). The resource p53 BAER (Binding and Expression Resource) provides the results of such a ChIP genomic study at UC Santa Cruz Genome Browser (Kent et al. 2002; Nguyen et al. 2018; Raney et al. 2014). These studies uncovered some surprising results. The most commonly found p53 target fragment consisted of the consensus binding site without any spacers, as expected. However, about a quarter did not contain even one recognizable half-site (Nguyen et al. 2018). Furthermore, the binding site fragments could be mapped back to their original position in the genome map. As a transcription factor, a reasonable expectation might be that the majority of the binding sites should be located proximally to a transcription start site. However, even using a generous 5-kbp distance cutoff definition for this, only 35% of the sequences satisfied the criterion. An additional 25% of the sites were intragenic, and 41% fell in intergenic DNA (Nguyen et al. 2018).

As a theoretical comparison, one may produce a model equipped to score a genome for p53 binding sites based on the extent of consensus homology present. One such typical model predicts on the order of 800,000 binding sites with a 20-mer binding site definition plus a spacer between half-sites of 0 to 15, or only one half-site (Menendez et al. 2009). Compared to the ChIP results, this is a gross estimation of binding sites. However, this also makes the important point that p53 is not just searching for the minimum consensus, but is somehow discriminating between the sites that apparently seem to match the consensus, but were not selected in the experiment. Thus, a complex picture of p53–DNA interactions emerges. The study raises the interesting question of why p53 appears to be predominantly preferring DNA sequences that do not have recognizable homology to the consensus and not binding in promoter regions. It hints that p53 may be recognizing more than just sequence and that the situation may be more complex.

### Indirect readout via L1 loop dynamics

As the evidence amasses, sequence homology alone can be sufficient for consensus sequences to be identified. However, for the majority of the sequences, this has not been the case, as we have argued above. An induced-fit mechanism could potentially be at work, and such an idea has been investigated for the L1 loop with Lysine 120 based on the differential conformations observed from the crystals (3Q05) mentioned above. Because the L1 loop is highly dynamic and two conformations are observed, the hypothesis that it could be a conformational switch as part of the greater recognition has been pursued (Lu et al. 2007; Petty et al. 2011). There are many energetically feasible conformations in dynamic interchange in the literature, which has also been our observation (Han et al. 2022; Ho et al. 2006; Joerger and Fersht 2007; Lukman et al. 2013; Slaw 2015). When the K120 contacts the DNA, it has the possibility to make two hydrogen bonds, contributing substantially to the enthalpy of binding at an interface with only 8 contacting residues expected as originally reported, and remains consistent with what we have seen in molecular dynamics (Han et al. 2022; Petty et al. 2011; Safieh et al. 2021; Slaw 2015). Petty et al. proposed an induced fit mechanism for this region and tested the hypothesis by measuring both the binding and unbinding kinetics. The expectation is that the binding affinity or by proxy the kinetic on-rate would be the driving factor for interaction that would differentiate between sequences. However, they report that the kinetic on-rate appeared indistinguishable between known p53 targets and p53-non-targets. They explained the anomaly via L1 loop dynamics. They surmise L1 loop dynamics play an integral role in the target sequence identification. Consistent with the kinetic off rate, the L1 dynamics aided in determining when p53 should disengage with the DNA. Thus, the overall scheme that arises from this view is that p53 indiscriminately binds onto DNA sequences. However, the operative question becomes whether it should remain bound; in other words, it is a time residency that becomes important. The dynamic L1 loop then is suggested by these experiments to probe the DNA to determine whether the p53 will remain and thus be a binding site, or disengage. The conformation of the DNA is a slightly bent shape could assist in the formation of the interaction, thus serving as an indirect readout component, as these researchers had initially set out to investigate. A scanning mechanism for p53 to locate its binding sites has been suggested by electron microscopy (Melero et al. 2011), and this interpretation seems consistent with that, considering that under normal cellular conditions, p53 is sequestered by MDM2. It is the cellular damage that releases p53 via MDM2 phosphorylation (Klein et al. 2021; Moll and Petrenko 2003; Momand et al. 2000; Thayer and Beyer 2016). This could pre-empt the apparent problem of ubiquitous p53 binding in the genome; when it is released, we may assume p53 binding is necessary because that is the condition of its release from MDM2, and otherwise, it is not present to do so at least in appreciable concentrations because it is bound by MDM2. When damage occurs, p53 quickly binds and scans the DNA, remaining at positions that either are binding sites by virtue of having excellent or close to perfect consensus, to optimize the enthalpic contribution, or binding by virtue of shape complementarity of the DNA or relative ease of the DNA to be deformed into such a conformation, with the assistance of the L1 loop training behind to potentially add up to 2 additional H-bonds to retain the p53 at the site.

### Additive energy model

We pushed an additive energy paradigm as proof of concept by constructing a computational model to attempt to quantitate the enthalpic contributions and estimate the corresponding contributions from indirect readout whether including the L1 loop or otherwise in an additive energy model (Thayer and Han 2017). The model numbers the positions of the direct contact region and uses the TG step at positions 5 to 6 in the binding site as a proxy for bending/bendability on the grounds that YpR steps are known from DNA mechanics studies to be highly flexible and easily bend (Beveridge et al. 2012, 2004; Dans et al. 2019; Dixit et al. 2005; Lavery et al. 2010; Pasi et al. 2014). For example, the approximately 90° bend in the cyclic AMP receptor protein structure (PDB ID 1CGP) is almost entirely attributable to the TG base pair steps exhibiting characteristic unstable stacking allowing them to bend (Schultz et al. 1991). A scoring scheme loosely based on free energy units in kilocalorie per mole was devised, and all possible decamers representative of one p53-DNA binding interface, or minimally required one half-site, were scored. The Smeenk et al. (2011) dataset was also scored, which provided 482 genomic binding sites.

The results suggested partitioning binding sequences into several categories depending upon the components of their scores, knowing that they were all in fact genomic sites. The first category and the single most populous contained sequences closely following the consensus. The 1TUP sequence falls into this category and thus may be considered an important structural insight for the canonical interactions. The second category represented those that lost the canonical TG step, and therefore the ability to readily bend at that position; they would have poor indirect readout due to a DNA kink, which may be recuperated by other features. AG was observed as the only allowable substitution in this category, and the C in the eighth position became conserved. The third class contained those with TG substituted by any other YR step, expected to have higher than average but lower than TG flexibility (Bertrand et al. 1998; Beveridge et al. 2004; Dixit et al. 2005), leading to higher presence of C at positions 4 and 8. The fourth category obliterated K120 and was compensated by the rise of homology among the group at several other positions. This group serves as an interesting counterpoint to the observations from the ChIP studies and those of Petty et al. (2011); they offer a representation of a sequence that would not necessarily be identified as a “consensus.”

The authors point out the possibility of an alternate H-bond formation when the YR step is lost to the YpR. The conservation of C4 is strictly enforced by introducing a G in the opposite strand to the base pair which could bind with R248. However, no direct evidence that this occurs is available. Interestingly, in simulations of wild-type p53, mutant p53, and p53 rescued by a small molecule, R248 emerges as one of several H-bonds in found exclusively in the Y220C mutant (Han et al. 2022). This ties back to the observations of Petty et al. that the kinetic on-rate was indistinguishable, but it was the kinetic off rate that discriminated between binding and non-binding sites. Consistent with that overall model, when the TG step is lost, the interactions with K120 seem to be conserved, except that category that could potentially pick up binding contributions from R28.

Overall, these experiments suggest that p53 binding is complex and potentially achievable by not only thermally accessible molecular configurational ensemble but also could be considered from a bioinformatics-based ensemble of interactions. By a variety of mechanisms, these energetic terms may sum up to sufficient binding interaction energy with a satisfactorily low kinetic off-rate, so as to allow a substantive residence time to activate transcription of target genes when a cell is experiencing damage and it has been released from MDM2.

### p53 isoform splice variants

**Fig. 3:**
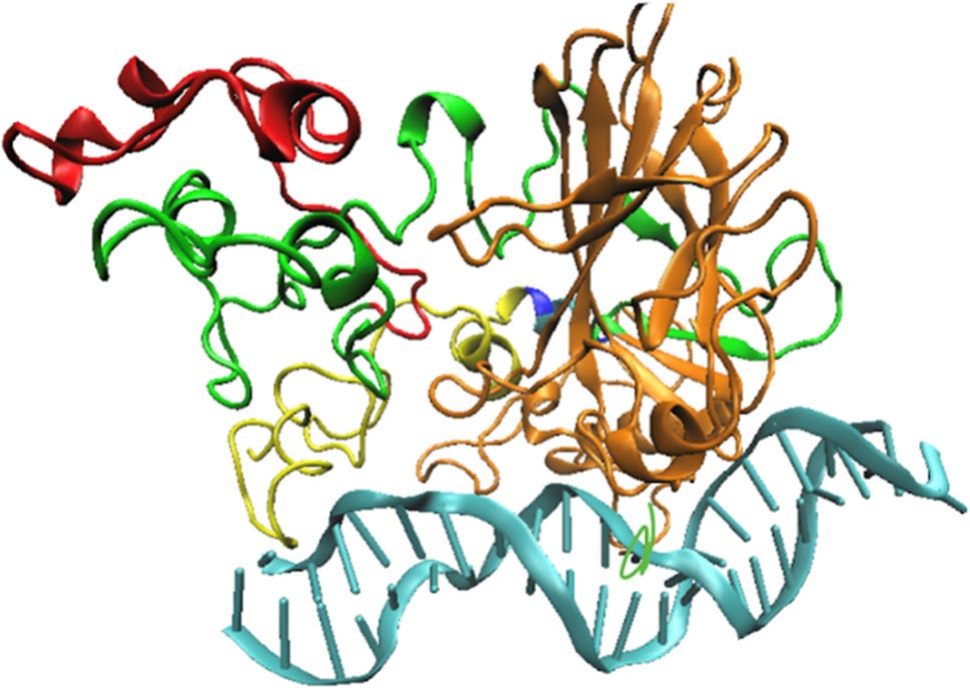
Full-length wild-type p53 protein showing isoform variable regions. The isoform variable regions are color-coded as follows: In red, residues 1 through 40. Green contains residues 41 to 132. Orange encompasses the DNA binding domain and the C-terminal, residues 133 to 340. Blue has residues 341 to 346, and yellow contains residues 347 to 393

Two additional factors, isoforms and p53 post-translational modifications (PTMs), may also regulate p53 interactions with DNA. Isoforms (Fig. 3) arise in eukaryotic gene expression when a multienzyme complex called the spliceosome edits an mRNA after it has been transcribed from the DNA but before it is translated into a protein (Wilkinson et al. 2020). In this way, the same DNA may encode several different protein sequences. Different isoforms of p53 have been found in different parts of the body, contributing to their differentiation (Bourdon 2007). The full length 393 amino acid variant is found in most tissues, yet some splice variants are tissue specific. For example, the splice variant Δ133p53ɑ is only found in the colon, bone marrow, testis, fetal brain, and intestine, and the variant Δ133p53ɣ is absent from the brain, heart, lungs, fetal liver, salivatory gland, breast, and intestine.

Human p53 has in total 12 isoforms with nine major forms, ranging from the full-length 393 amino acid wild-type to the much smaller residue number 94–312 fragment which approximately corresponds to the crystallized DBD fragment. The DBD is present in all isoforms; the isoforms vary in the truncations of the N and C terminal disordered regions. Isoforms have been suggested to play a role in cancer development (Bourdon 2007; Bourdon et al. 2005; Steffens Reinhardt et al. 2023). To begin to understand the mechanism of how this might arise at the molecular level, MD simulations of a selection of isoforms indicated that the structural behavior of the DBD particularly with respect to its interaction with DNA was modulated by the different isoforms through an allosteric mechanism, since the changes occur in the intrinsically disordered regions and not directly in the DBD. This suggests that the splice variants contain a hard-coded regulation that may contribute to the differences in p53 behavior in different tissues, which may be related to the differences in tumor prevalence depending on where in the body they arise (Armour-Garb et al. 2022).

### p53 PTMs

P53 has been likened to a decision-making circuit board integrating information about the cell status from multiple enzymes reporting on the cellular status (Sullivan et al. 2012). Events compromising the genetic material, such as DNA double-strand breakage, formation of thymidine dimers by irradiation, hypoxia, heat shock, and exposure to radioactive chemicals, lead to the activation of p53. A mechanism by which this occurs is the direct modulation of p53 activity and specificity by PTMs (Joerger and Fersht 2008; Okorokov et al. 2006). PTMs are covalently attached (or removed) functional groups transferred by enzymes to specific residues in the protein, mainly in the N and C terminal regions. The most common PTMs are phosphorylation, methylation, acetylation, and ubiquitination. Several recent reviews provide an overview of PTM effects (Appella and Anderson 2001; Gu and Zhu 2012; Sullivan et al. 2012). Gu and Zhu provide a comprehensive diagram summarizing all currently known PTMs organized by predominant function. Once p53 becomes activated, it prevents the transmission of genetic errors to new cells by inducing cell cycle arrest and DNA repair, or, when cells are beyond repair, through initiating apoptosis. As a transcription factor (Bourdon et al. 2005), it carries out this role of determining cell fate by binding in the promoters of target genes associated with those outcomes and regulating their transcription (Okorokov and Orlova 2009). PTMs exert regulatory control over the sequence specificity of p53 in a complex combinatoric code on the N and C termini (Gu and Zhu 2012; Smeenk et al. 2011) contributing to the cell’s fate, but the molecular mechanism by which this occurs is not known. Furthermore, the binding sites of the two pathways are not separable on the basis of the DNA sequence. Thus, understanding the mechanism of PTM control will shed light on how the cell fates are determined and provide insight into how this might be manipulated by therapeutics, thereby inducing the eradication of tumors.

Ten PTMs independently capable of regulating promoter-specific enhancement exist in the combinatorically complex PTM regulatory scheme, and offer the simplest starting point for understanding how PTMs affect DNA binding specificity. Of these, eight (S46, K320, R333, R335, R337, K370, K373, K382) are located in the IDR and thus can provide information on the allosteric nature of the regulation. From this list, we have selected to showcase information on S46 and K320 to represent both a phosphorylated and an acetylated residue, and to include a residue in each of the disordered regions. A broad literature on PTMs exists; within the scope of this review, we will glean general ideas from these well-defined examples.

Acetylation of the C-terminal K320 (Ac-K320) is associated with activation of the high-affinity p53 target sequence p21/WAF that mediates cell cycle arrest. The acetylation occurs via a cascade in which the kinase HIPK2 interacts with PCAF, a histone acetyltransferase (HAT) also capable of modifying p53, to increase its acetylation activity of p53 at K320 (Di Stefano et al. 2005). p53 has been shown to bind in the p21 promoter to two distinct upstream binding sites (− 2.27 kb termed the 5′ site and − 1.38 kb termed the 3′ site) by DNaseI chromatin footprinting (Espinosa and Emerson 2001). Ac-K320 p53 binding specifically with this promoter’s sequence up-regulates the expression of the p21 gene product (Di Stefano et al. 2005; Knights et al. 2006).

Phosphorylation of the N-terminal S46 (P-S46) is associated with activation of the p53 target gene, apoptosis-inducing protein p53AIP1, and commits the cell to apoptosis (D’Orazi et al. 2002; Oda et al. 2000). In response to ultraviolet irradiation, HIPK2 kinase transfers a phosphate to S46. Under the alternate stress of IR irradiation, the protein ATM activates dual-specificity tyrosine-phosphorylation-regulated kinase 2 (DYRK2) which phosphorylates S46 of p53 (Saito et al. 2002; Taira et al. 2007; Yoshida 2008). P-S46 activates p53 to bind to the promoter of p53AIP1, increasing the production of p53AIP1 protein, which initiates a mitochondrial apoptotic pathway (Matsuda et al. 2002; Oda et al. 2000; Yuan et al. 2012).

PTM modification of p53 N and C terminal regions to control DNA binding specificity is an example of allosteric regulation. Allostery occurs when an effector molecule binds and causes a change in a site in a distant part of the protein (Berezovsky and Nussinov 2022; Cooper and Dryden 1984; Fenton 2008). The phenomenon was first recognized in hemoglobin half a century ago (Monod et al. 1963) and has become so widely recognized in biological processes that it is termed “the second secret of life” (Fenton 2008). Over the years, the definition has broadened to include phenomena such as covalent allosteric effectors; PTMs constitute an important example of this type. Thus, in the allosteric view of the system, the effector is a PTM, and the active site modification is the changed binding affinity between the protein and its DNA binding sites.

### On allosteric drug design

We have at this point considered the overview of p53 structure, which is most clear at the monomeric level, and considerably more controversial at higher order. Biochemical experiments studying the binding sequences of actual p53 sites paint a picture of a protein that readily recognizes its consensus sequence which binds as shown in the original 1TUP structure, but the situation quickly becomes more complex when considering the myriad of biological DNA sequences binding well and apparently in a discriminatory way that do not properly follow the consensus. From the bioinformatic point of view, the problem is considerably underdetermined, with at best three, or perhaps only two, of the contacts conferring specificity to the site. The idea that binding may occur by indirect readout taking into account the structure and dynamics of the DNA itself thus becomes an interesting proposition. Considering the variations that may occur due to post-translational modifications and various isoforms of p53 raises the idea of even more possibilities for factors controlling the p53-DNA interaction, which are very much in the stages of still being sorted out. Nevertheless, we emerge with the idea that p53 in all these cases binds DNA at the original interface. The Y220C rescue provides the case in point that molecular rescue is not only plausible, but also possible despite the complexities.

Encouraged by the Y220C success, we now turn our attention to a critical problem raised at the outset, namely, how to tackle designing drugs to restore p53 native activity. The serendipitous discovery of allosteric regulators such as the p53 restorative compound PK11000 has opened the idea of de novo design of allosteric regulators of proteins in which the active site is controlled from a distance. These will have a unique role in situations where one wishes to modulate the active site activity while leaving it fully intact. One apt application of this idea is in the reactivation of mutant proteins.

Y220C rescue by PK11000 operates on the principles of restoring a reasonable number of H-bonds at the active site (Han et al. 2022). This is of course much easier to arrive at post priori with the solution in hand. The very fact that it was not designed from first principles leaves researchers at a loss for how to improve upon an excellent idea such that more iterations could be attempted with reasonably good guesses. Since the active site constitutes the protein-DNA interface, we are faced with the difficult problem of designing drugs that act upon this interface remotely. Any researcher hoping to design a drug to restore WT function must reverse engineer allosteric interactions, a difficult task indeed. While humans have limited success at this, machines are considerably better when a sufficient dataset and proper training are available, and when one has a specific outcome or goal that can be quantitated in a way that can be presented to a machine. In the second half of the review, we report on ideas for the identification of allosteric sites of control and the means by which machines may be implemented to identify patterns to optimize through making moves equating to manipulating branches of graphs representing molecules. We propose exciting advances and ideas that, although have not yet realized the molecules needed, provide a way forward in directions that have heretofore not been possible.

### Machine learning in allosteric drug design

Generative adversarial neural networks (GANNs) (Aggarwal et al. 2021; Arora et al. 2023; Creswell et al. 2018) represent a powerful framework for unsupervised learning and generative modeling in machine learning. Comprising two distinct neural networks, a generator and a discriminator, GANNs operate through an adversarial training process. The generator network aims to generate synthetic samples, in this case a drug molecule, that resembles real data, while the discriminator network learns to differentiate between real and synthetic samples. Through an iterative training process, the generator seeks to improve its ability to generate realistic samples, while the discriminator simultaneously enhances its discriminative capability. Using a graph representation of the drug, a move for improvement of the drug equates to a change in the graph subject to rules of basic chemistry. This competitive interplay fosters the refinement of both networks, leading to the generation of increasingly realistic and high-quality synthetic samples. GANNs have demonstrated remarkable success in various domains, such as image synthesis, text generation, and audio generation, making them a widely researched and influential methodology in contemporary machine learning research.

GANNs have emerged as a promising tool in the field of drug design and drug discovery (Abbasi et al. 2022; Bian and Xie 2021; Padalkar et al. 2021; Tripathi et al. 2022). They offer a unique approach to generating novel molecules with desired properties by combining the power of deep learning and generative modeling (Bian and Xie 2021). The generator network in GANNs can be trained to generate new chemical structures, while the discriminator network assesses the quality and desirability of these generated molecules based on various criteria, such as drug-likeness (Guan et al. 2019), bioactivity (Gaulton et al. 2012; Lane et al. 2021), or synthetic feasibility (Gao and Coley 2020; Liu et al. 2022; Thakkar et al. 2021). The cooperative training between the generator and discriminator enables GANNs to learn and capture the underlying chemical space, leading to the generation of novel molecules that possess desirable drug-like properties. GANNs hold great potential in accelerating the process of lead optimization, hit identification, and de novo drug design by facilitating the exploration of vast chemical spaces and enabling the discovery of novel therapeutic candidates with improved efficacy and safety profiles (Abbasi et al. 2022; Padalkar et al. 2021). Despite the challenges in optimizing the balance between novelty and drug-likeness, GANs offer a promising avenue for the discovery of new drugs and the design of targeted therapeutics in an efficient and data-driven manner.

Molecular docking (Bai et al. 2023; Gschwend et al. 1996; Meng et al. 2011; Pinzi and Rastelli 2019) is a computational technique in which small molecules are placed and optimized in a binding pocket in a larger molecule, most often a protein. Autodock Vina (Trott and Olson 2009) is an open-source docking implementation maintained by the Forli lab at The Scripps Institute. It is one of the fastest and most widely used open-source docking engines. It uses a simple scoring function, contributing to its speed and ease of use. Flexible docking, which enables the user to specify a subset of the system to undergo coordinate optimization during the docking process in response to the inherent force field, yields the most precise results. This, however, comes at a steep computational cost. Autodock Vina takes advantage of parallelization of calculations as well as the ability to process jobs on graphical processing units (GPUs) (Gawehn et al. 2018; Pandey et al. 2022), enabling speedups of orders of magnitude over previous generations of docking software such as Autodock 4. Thus, researchers can now access high-precision docking in a shorter amount of (wallclock) time, or utilize the speedup to increase the number of compounds screened computationally.

Experimental screening of compounds, known as high-throughput screening (Liu et al. 2022; White 2000), has been the standard for drug discovery of biologically active hits. The high monetary cost of such screens is prohibitive, leading to the adoption of aforementioned in silico screening methods, of which docking is one of the most prominent. While docking screens are most often used to screen a pre-existing virtual library of compounds, it can just as readily be used for screening and assessing compounds derived from generative AI approaches. Computational approaches using virtual libraries are an attractive way to screen an expanded chemical space. Presently, enumerating tens of billions of molecules is within the realm of possibility. They, however, are unlikely to exist from natural sources and thus require synthesis. Few can actually be made due to constraints of time, storage capacity, and cost of materials. Docking, however, can be instrumental in providing a rank order to indicate which are most promising (Shen et al. 2023). The two major concerns regarding docking are that the molecules may not be readily synthesizable, and accuracy of docking scores historically has not been sufficiently accurate to identify true hits, mostly due to false positives which are magnified especially in such a large space. However, recent advances have mitigated these problems (Cavasotto and Di Filippo 2023). While the approach remains imperfect, it has made this option viable for many labs such that it is of practical use. The first is the number of “make-on-demand” libraries (Schmidt et al. 2022) offered by vendors and academic laboratories. Users may request a molecule of interest and a dedicated team will then synthesize it based on 2- or 3-component reactions and purify the compound. Making use of the power of combinatorics, the company Enamine, for example, offers some 140 reactions among 120,000 molecular building blocks to offer the ability to synthesize a library spanning a space of 29 billion molecules, greatly increasing the probability that a desired molecule of interest can be created on demand for a reasonable price.

While molecular docking continues to deal with known imperfections, it has proven to be successfully implemented to reasonably prioritize molecules worthy of subsequent study even in these newly accessible ultra-large libraries even in the tens of billions of size range. Integrating docking scores into the generator’s scoring function is a simple yet powerful way to improve the quality of results generated. No longer are the molecules generated in a vacuum, but they are generated and iteratively improved within the specific context of p53 and its DNA interactions.

### Allosteric p53 and allosteric drug design

To begin this portion, we foray into the topic of allostery and its role in drug design. Allosteric regulation of proteins is a strategy to finely modulate protein activity where the active site is fully intact and able to perform function (Guarnera and Berezovsky 2020; Peng 2015). Allostery, or action at a distance, has been widely regarded as so pervasively ubiquitous to biology that it has been dubbed “the second secret of life,” second only to the DNA genetic code itself (Fenton 2008). Such a signal can be transmitted even up to 100 angstroms away (Jayaraj et al. 2023; Lakhani et al. 2017). The p53 protein has been identified as an allosteric protein (Degn et al. 2022), and the hotspot mutation Y220C that has been implicated in allosteric drug design appears far from the DNA binding interface (Bauer et al. 2016). Allosteric drugs operate orthosterically by binding distant from the active site, enabling it to modulate activity remotely rather than occlude activity (Nussinov and Tsai 2012). Allosteric drugs may be more highly selective to their target because allosteric points of regulation are likely free from evolutionary selection, affording variation even among proteins in the same family (Lakhani et al. 2017). Allosteric effectors also often modulate the level of activity as opposed to completely turning activity on or off (Guarnera and Berezovsky 2016). This allows for safer dosage, especially for targets carrying out essential functions, and may make drugs more safe for accidental overdose or ingestion. Furthermore, because the allosteric site differs from the active site, the drug does not compete with the native ligand, and therefore lower dosages may be effective (Grover 2013). Furthermore, allosteric drugs open possibilities for drugging previously undruggable targets (Hantschel et al. 2011; Hassin and Oren 2023). While drugging an active site provides only a single binding site, many allosteric points of control may be available, offering many new inroads to successfully drugging these targets.

Although allosteric drugs offer numerous advantages, they continue to be discovered most often by compound screens, which are costly and require additional experimentation to determine how and where they bind (Chatzigoulas and Cournia 2021; Guarnera and Berezovsky 2020). Additionally, an assay for the binding of the allosteric effector and a functional assay will be required. Even at the cost of $1 per drug, likely a gross underestimation, a typical screen involving 1 million compounds would cost $1 million, and if anything is even found, there is little to no mechanistic knowledge available without further experimentation for refinement as is often required as testing in animal models and clinical trials often reveal. Thus, computational approaches for computer-aided drug design (CADD) are an indispensable strategy for researchers both in the industrial and academic setting (Durrant and McCammon 2010). CADD approaches have enjoyed wide success in development of drugs targeting active sites and other direct points on proteins (Hernández Alvarez et al. 2019; Irsheid et al. 2019; Kim et al. 2017; Ogunlana et al. 2022; Wang et al. 2022).

### Sectors: identifying allosteric points of control for drug design

One formidable challenge to allosteric drug design is to identify locations of allosteric points of control. Advances have been made to identify allosteric points of control (Lakhani et al. 2020), a foremost model having been based on the idea of sector analysis. The “sector hypothesis” puts forth the idea that sector residues convey allosteric information between the site of the allosteric binding and the active site. The idea originated with making use of the sector analysis of the stock market arena to identify residues illustrating covariance in proteins over evolutionary time. Sectors convey the idea that groups of covarying entities may have some predictive capabilities for a larger trend, one of which may be allosteric signaling, an idea that was supported by early sector implementation with experimental verification (Lakhani et al. 2020; Reynolds et al. 2013, 2011).

The next generation of sectors was built on the idea of identifying residues of a single protein that covary due to thermal fluctuations in molecular dynamics simulations, called MD sectors. Thus, the covariance from multiple sequence alignments can be exchanged for covariance from MD simulation trajectories, removing the need to work with proteins from many species. It also provides a handle to engage with statistical mechanics and population dynamics by dividing the snapshots of a well equilibrated system into various clusters and following their interchange, alongside the identification of the sector residues. The MD sectors lend key insights into allosteric signaling, a perturbation at one locale that produces an effect at a distal site (Lakhani et al. 2020, 2017). The definition of allosteric may be aggrandized to encompass distant point mutations affecting a binding site and the binding of a small therapeutic molecule. Thus, MD sectors provide a quantifiable method to analyze allosteric signals which enables the engineering of allosteric regulators to effect a desired outcome in tweaking the activity of an active site from a distance. Analyzing MD enables the ability to interpret not only the covariance of individual residues, but also grouping the highest 20% of covarying residues into sectors.

MD sectors have recently been computed on p53 with an eye towards understanding how point mutations might propagate through the protein to affect the active site (Fabry and Thayer 2023). Recent iterations of MD sectors display that residues analyzed from MD trajectories can be quantified into a sector. In the case of research into the p53 tumor suppression protein, the ability to quantify sectors and visualize how allosteric signals correspond to one another becomes a vital area of study. In the latest study and development of new MD sector algorithms, the potential for quantifying multiple sectors within one protein has become a main subject of research. One sector displays a region of allosteric signals within a protein; however, the introduction of dividing a single protein into a network of multiple sectors, each hosting their own system of correlated residues, provides the potential for a continued deeper analysis of well-studied and newer unresearched proteins alike.

In addition to using MD sectors to model allosteric networks, recent work suggests that allosteric networks can also be captured well by a new method called heat kernel analysis (HCA) (Avramidi 1999). The method borrows the idea of flow from physics; instead of following the flow of heat in coupled bodies, we instead use the framework to study the flow of the allosteric signal through protein residues in a graph-theoretic framework. Kernels are mathematical operators that perform a transformation to maximize the separation of some signal of interest in a latent space. We combine HCA with deep generative adversarial neural networks to generate small molecules. Machines modify docked molecules to restore native p53 conformation dynamics profiles from molecular dynamics simulations.

The heat kernel itself is a fundamental solution to the heat equation, representing the distribution of heat at any given point in space and time. The solution to the heat kernel for a given time *t* is given by the equation:

$${h}_{t} = {P}_{e}^{-tLPT}$$

where *P* is the matrix of eigenvectors of the normalized graph Laplacian, and *e*^−*tL*^ is the diagonal matrix with *e* raised to the power of each eigenvalue of the graph Laplacian multiplied by *t*. Analyzing the heat kernel using eigendecomposition, we can gain insights into various aspects of diffusion processes. Eigendecomposition is the factorization of a matrix into a canonical form, whereby a diagonalizable matrix is represented in terms of its eigenvalues and eigenvectors. The heat kernel provides information about the probability density function of the diffusion process, revealing how the distribution of heat changes over time and space. In this context, “heat” represents the allosteric signal diffusing over the protein as captured by the interaction energies from MD simulations, of which electrostatic has emerged as the most significant.

Given that any drug that is able to rescue mutated p53 will almost certainly be allosteric, identifying what the allosteric network is becomes the first-order problem. This approach of using MD simulations and a combination of sector analysis and HCA to elucidate the allosteric sectors within the protein offers invaluable insight into how various mutations and drugs alter the allosteric network, and what combinations of mutations and drugs offer WT rescue (Abramson 2021; Cowan 2023).

### Drug design summary

The above two sections thus define a straightforward (and admittedly idealistic) drug development pipeline. Drugs are generated using a generative machine-learning network, which is constantly assessing generated molecules on their docking abilities with p53 using Autodock Vina. Then, simulations are run with the drug bound to the mutant form of the protein, and using MD sector analysis and heat kernel analysis, the allosterically connected residues are identified and compared to those of the wild-type. The process then repeats, until a sufficiently well-performing drug is generated.

## Conclusion

We have seen that the p53-DNA interactions are complex and nuanced; it can bind by virtue of a consensus binding site, or something far more complex involving indirect readout of the dynamical properties of the DNA itself. Furthermore, tetramerization, post-translational modifications, and the presence of isoforms all contribute to the life or death decisions p53 is involved in.

Turning then to prospects of molecular cures for cancers, we review the progress in light of the many new advances available to expand the possibilities beyond the status quo, which largely rely upon educated chance. Recognizing that both mutations and small molecules may act from a distance, drawing upon knowledge of allostery greatly informs new approaches. Sector analysis may assist researchers in identifying allosteric residues. While rescue does not guarantee the recapitulating the binding interface, recent work focused on allosteric sectors demonstrates a promising role in explaining how the serendipitously discovered PK11000 works, alongside the potential to analyze other drugs focused on p53. Although PK11000 itself has not been found suitable for human use, new insights provide ideas as to how the experimental drug could have been engineered, so that we may design other trials de novo.

Furthermore, the new possibility of designing drugs in silico opens new prospects. Potential small molecule therapeutics can be represented utilizing graph theory and machine learning techniques such as general adversarial neural networks. Such methods may be able to utilize feedback from molecular simulations, energetics, or other related data to modify those graphs, thereby engineering allosteric molecules based on feedback through the network. Challenges involve developing and tuning pipelines to provide feedback from MD simulations, allostery, the H-bond interface, DNA binding interface, sectors, and any other computational metric in a feedback loop to improve drugs. While utilizing computational techniques to their fullest extent has not yet been realized, the method holds great promise for advances in the development of molecular therapeutics. Furthermore, computational methods are often translational, with potential applications to other currently undruggable targets. Such means may also have applications for engineering molecules of a desired function.

In conclusion, p53 is a complex, fascinating, and timely molecule to study. Some 3 decades after its structural discovery, we have yet to find molecular cures to restore its mutants to native function. Combining biophysical understanding with machine learning, new horizons are now opening on approaches with potential to shed greater light on its biomolecular function while making an impact on finding molecular cures for cancer.

## Data Availability

Not applicable. This article is a review article.
